# Chimeric yellow fever 17D-Zika virus (ChimeriVax-Zika) as a live-attenuated Zika virus vaccine

**DOI:** 10.1038/s41598-018-31375-9

**Published:** 2018-09-04

**Authors:** Maryann Giel-Moloney, Ana P. Goncalvez, John Catalan, Valerie Lecouturier, Yves Girerd-Chambaz, Fernando Diaz, Francisco Maldonado-Arocho, Raul C. Gomila, Marie-Clotilde Bernard, Ray Oomen, Simon Delagrave, Nicolas Burdin, Harold Kleanthous, Nicolas Jackson, Jon Heinrichs, Konstantin V. Pugachev

**Affiliations:** 1 Sanofi Pasteur Research & Development, Cambridge, MA USA; 2 Sanofi Pasteur Research & Development, Marcy-l’Étoile, France; 3 Sanofi Pasteur Research & Development, Swiftwater, PA USA; 4 Present Address: VL46 Inc., Cambridge, MA USA; 5 Present Address: Kanyos Bio, Cambridge, MA USA

## Abstract

Zika virus (ZIKV) is an emerging mosquito-borne pathogen representing a global health concern. It has been linked to fetal microcephaly and other birth defects and neurological disorders in adults. Sanofi Pasteur has engaged in the development of an inactivated ZIKV vaccine, as well as a live chimeric vaccine candidate ChimeriVax-Zika (CYZ) that could become a preferred vaccine depending on future ZIKV epidemiology. This report focuses on the CYZ candidate that was constructed by replacing the pre-membrane and envelope (prM-E) genes in the genome of live attenuated yellow fever 17D vaccine virus (YF 17D) with those from ZIKV yielding a viable CYZ chimeric virus. The replication rate of CYZ in the Vero cell substrate was increased by using a hybrid YF 17D-ZIKV signal sequence for the prM protein. CYZ was highly attenuated both in mice and in human *in vitro* models (human neuroblastoma and neuronal progenitor cells), without the need for additional attenuating modifications. It exhibited significantly reduced viral loads in organs compared to a wild-type ZIKV and a complete lack of neuroinvasion following inoculation of immunodeficient A129 mice. A single dose of CYZ elicited high titers of ZIKV-specific neutralizing antibodies in both immunocompetent and A129 mice and protected animals from ZIKV challenge. The data indicate that CYZ is a promising vaccine candidate against ZIKV.

## Introduction

Zika virus (ZIKV) is a flavivirus transmitted primarily by *Aedes* spp mosquitoes. Genetically it belongs to the Spondweni group of flaviviruses which is distinct from the four main flavivirus serocomplexes: yellow fever, Japanese encephalitis (JE), dengue, and tick-borne encephalitis (TBE)^1^. ZIKV was first isolated in Uganda in 1947 from a febrile Rhesus monkey, and before 2007 circulated only in Africa and Asia, with very few described cases of mild, dengue-like disease in humans. Clinical manifestations included fever, malaise, headache, dizziness, anorexia, retro-orbital pain, and maculopapular skin rash. However, recent spread of the virus to the Pacific and the Americas has been accompanied by an unprecedented increase in transmission rates and has linked ZIKV infection to microcephaly and other birth defects in infants of mothers infected during pregnancy as well as neurological disorders in adults, mostly Guillain-Barré syndrome (GBS)^2^. In addition to transmission by mosquitoes which accounted for the vast majority of infections, sexual transmission has been well documented^3^.

The first ZIKV outbreak outside of Africa and Asia occurred in 2007 on Yap Island, Micronesia with 919 cases (affecting 18% of the island population) of mild disease. This was followed by an outbreak in French Polynesia in 2013–2014 with approximately 8,750 cases of symptomatic infection, including 42 cases of GBS accompanied by encephalitis, meningitis, paraesthesia, facial paralysis and/or myelitis. Brazil reported its first Zika case in May 2015 and the outbreak peaked in early 2016 with an estimated 1.3 million cases of disease and approximately 3,500 suspected microcephaly cases including 46 deaths in 20 states^4^. ZIKV has subsequently spread to other Latin American countries and to the southern continental U.S. Despite a recent decline in cases, ZIKV remains a significant emerging pathogen^5^.

As with other flaviviruses, ZIKV is a small (50 nm in diameter), enveloped, positive-strand RNA virus^6^. Flavivirus particles contain a nucleocapsid composed of viral RNA and capsid protein (C) surrounded by a lipid envelope containing the envelope glycoprotein (E) and membrane protein (M). The genomic RNA (~11,000 nucleotides in length) encodes a single open reading frame (ORF) flanked by 5′ and 3′ untranslated regions (UTRs). The ORF is translated into a polyprotein precursor encoding three structural proteins (C, prM precursor for mature M, and E) and nonstructural (NS) proteins NS1–NS5 required for virus replication in the cytoplasm of infected cells. The polyprotein is cleaved by cellular and viral (NS2B/NS3) proteases to yield individual proteins. Progeny virions bud into the lumen of the endoplasmic reticulum (ER) and are transported to the cell surface through the exocytosis pathway^7^. The E protein is the main immunogen eliciting neutralizing antibodies that are considered to be the primary correlate of protective immunity against flavivirus infection. Virus-specific cytotoxic T-lymphocyte (CTL) response is the other key attribute of immunity. Multiple CD8+ and CD4+ CTL epitopes have been characterized in various flavivirus structural and non-structural proteins^8^. All documented ZIKV strains represent a single serotype and thus a vaccine based on one strain should be effective against other circulating strains.

Flavivirus vaccines which have been in use for decades are either live attenuated vaccines (LAV) obtained by empirical attenuation (17D vaccine against YF and SA14-14-2 vaccine against JE) or inactivated virus vaccines (INV) available for JE and TBE^9^. INVs require several doses for priming, followed by periodic boosters to maintain immunity, while the main advantage of LAVs is that they generally require very few doses to elicit durable protective immunity without the need for adjuvant. For example, YF 17D is considered one of the strongest immunogens ever developed, providing protection after a single dose that is believed to be life-long. It has demonstrated safety for children beginning at 9 months of age and for adults, with serious adverse events occurring extremely infrequently. Based on theoretical concerns, YF 17D vaccination is not recommended during pregnancy and breastfeeding, but according to the WHO it can be administered during YF epidemics associated with high transmission risk. Large numbers of pregnant women have been vaccinated in the past without any evidence of adverse effects for the fetus^10^.

Sanofi Pasteur has developed a novel chimeric LAV platform, ChimeriVax, targeting dengue, JE and West Nile (WN) viruses. ChimeriVax-JE and -DEN vaccines are now licensed human vaccines (IMOJEV™ and Dengvaxia®). ChimeriVax vaccines are constructed by replacing the prM-E envelope protein genes in the YF 17D genome with heterologous flavivirus prM-E genes, resulting in highly attenuated and immunogenic chimeric viruses with altered antigenic specificity. A number of studies in animals and humans have demonstrated the safety and immunogenicity of ChimeriVax-based vaccines. In addition, similar to YF17D virus which has lost its ability to be transmitted by mosquitoes, ChimeriVax vaccines are not transmitted by mosquito vectors^9,11,12^.

Various strategies are being pursued to develop an effective vaccine against Zika, such as INVs, LAVs, virus vectored, recombinant protein, and nucleic acid based candidates^5,13–17^. Because a Zika vaccine must be safe for pregnant women and their fetus, currently the preferred approaches are those that do not rely on replicating virus. Nevertheless, depending on LAV characteristics as well as future ZIKV epidemiology, a LAV could become preferable, e.g., if a LAV is proven to be safe for pregnant women or for universal vaccination of children before they are sexually active to elicit long-lasting immunity after a single dose, similar to the rubella live vaccine. Here we constructed a ChimeriVax-Zika (CYZ) candidate that efficiently replicates in Vero cells (an established substrate for LAV production), and evaluated its attenuation and tissue tropism compared to wild-type (wt) ZIKV, and its immunogenicity and protective efficacy in both *in vitro* and murine models.

## Results

### Construction of ChimeriVax-Zika (CYZ) candidates

**Figure 1:**
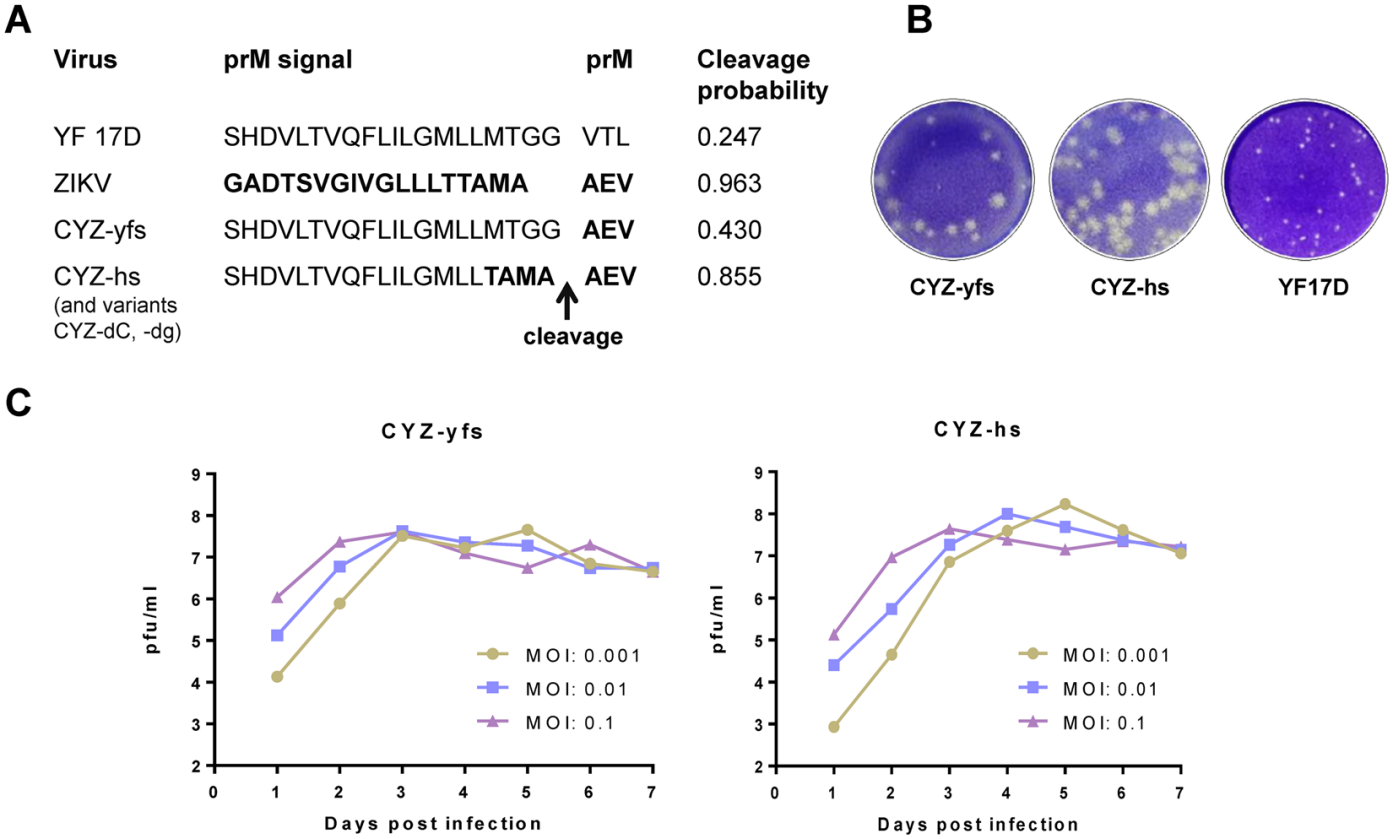
CYZ-yfs and CYZ-hs (lead) candidates. (**A**) Predicted signalase cleavage scores by SignalP3.0 software for parental viruses, YF 17D and wt ZIKV, and the CYZ chimeras. ZIKV-specific residues are in bold. Several additional CYZ variants were constructed containing additional attenuating genetic modifications, such as CYZ-dC (3-amino acid deletion in C) and CYZ-dg (de-glycosylated E protein) variants based on CYZ-hs (see text for details). (**B**) Plaque morphologies of CY-yfs, CYZ-hs, and YF 17D parent virus in Vero cells. Plaques were stained with Crystal violet on day 5 post-infection. (**C**) Growth curves of CYZ-yfs and CYZ-hs viruses in serum-free Vero cell culture at indicated MOIs.

CYZ vaccine viruses were constructed using the prM-E gene cassette of the French Polynesia-2013 ZIKV strain, which was selected based on phylogenetic analysis demonstrating that this strain belongs to the Asian-American lineage of ZIKV^18^ and because its prM-E amino acid sequence is identical to recent strains isolated in Brazil (2015), Haiti (2014), Martinique (2015) and Puerto Rico (2015). A CYZ-yfs variant was generated which contains the ZIKV prM-E genes inserted into the YF 17D backbone in the same fashion as in other previously constructed ChimeriVax vaccine viruses^9^, between the C/prM and E/NS1 signalase cleavage sites. Thus, the signal sequence for prM protein in this virus is derived from YF 17D. Similar to YF 17D virus, cleavage probability at the C/prM signalase site of CYZ-yfs is relatively low as determined with the SignalP3.0 program^19^ (http://www.cbs.dtu.dk/services/SignalP-3.0) (Fig. 1A). It has been proposed that inefficient signalase cleavage at the C/prM site is a prerequisite of efficient replication of flaviviruses based on studies using YF 17D and Murray Valley encephalitis viruses^20–22^. Interestingly, cleavage probability at this site of wt ZIKV was found to be significantly higher than YF 17D (Fig. 1A). To evaluate how increasing the predicted cleavage efficiency would affect replication of the chimera, a CYZ-hs variant was constructed containing a hybrid YF 17D/ZIKV prM signal which increased the cleavage probability to that of ZIKV (Fig. 1A). Unexpectedly, growth curve analysis showed that while both chimeras replicated efficiently in Vero cells, the CYZ-hs variant generated several-fold higher peak titers [≥8 log_10_ plaque-forming units (PFU)/ml at multiplicity of infection (MOI) of 0.01 and 0.001 PFU/cell] compared to CYZ-yfs (Fig. 1C). Both variants formed clear plaques that were larger than plaques of YF 17D parent (Fig. 1B) and were shown to be genetically stable by sequencing at P2 (research stocks) and P5 passages. Control chimeras designated YF/Senegal-1894 and YF/Cambodia-2010 were also constructed with prM-E genes from African and Asian ZIKV strains, Senegal-1984 (DAKAR 41519) and Cambodia-2010 (FSS 13025), containing the YF 17D-specific prM signal sequence. These strains are unrelated to the recent outbreaks in the Pacific and Americas. The resulting chimeras replicated as efficiently as CYZ-yfs in Vero cells (data not shown).

Because the attenuation profiles of CYZ-yfs and CYZ-hs chimeric viruses were unknown, other CYZ variants were also engineered in parallel with additional attenuating modifications including 3 amino acid changes in the E protein corresponding to the 3 attenuating JE SA14-14-2 vaccine-specific changes that had been incorporated in the ChimeriVax-WN02 vaccine (E-107 L to F, E-316 A to V and E-440 K to R^23,24^, an Asn to Gln mutation at E-154 residue ablating the N-linked glycosylation motif Asn-X-Ser/Thr in the E protein (variant CYZ-dg; based on CYZ-hs with hybrid prM signal), and small deletions in the YF 17D-specific C protein (variant CYZ-dC with residues PSR deleted at amino acids 40–42 of the C protein; based on CYZ-hs) and 3′UTR (deletion CAGGT at nts 256–260 of the 3′UTR). Other modifications in these genome elements have been evaluated for their ability to increase attenuation of various flavivirus vaccine candidates^16,25–29^. Deglycosylation of the E protein in the context of whole wt ZIKV was recently shown to confer some attenuation, which however was deemed insufficient for a safe vaccine candidate^30^. The additionally constructed CYZ variants were examined for replication in Vero cells (generally they grew to titers up to 10-fold lower compared to the parental CYZ chimeras) and some for attenuation/immunogenicity *in vitro* and *in vivo* (e.g., see below, and data not shown).

### CYZ attenuation (after IC inoculation) and immunogenicity in immunocompetent mice

**Figure 2:**
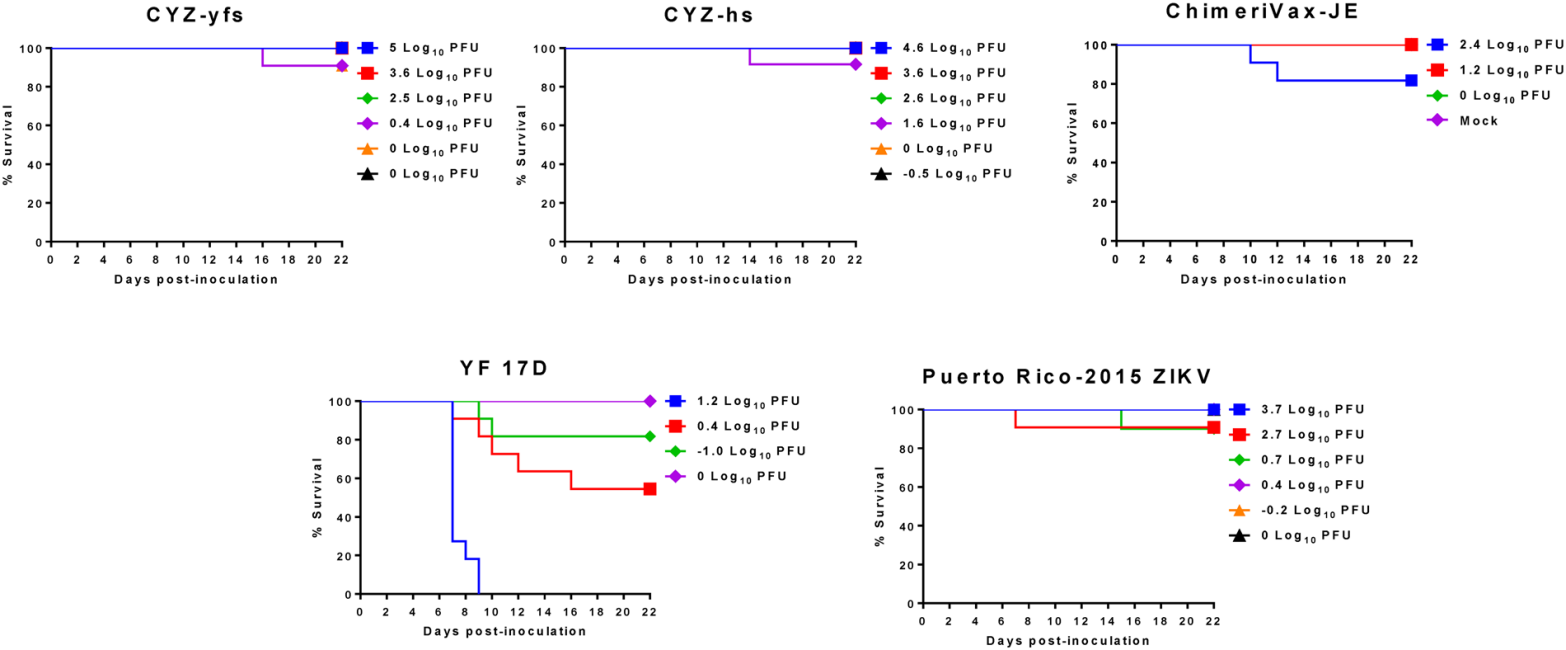
Attenuation of CYZ-yfs and CYZ-hs variants in suckling mouse neurovirulence test compared to YF 17D vaccine benchmark control. 5-day old ICR suckling mice were inoculated IC with graded doses of viruses (the intended doses were −1 to 4 log_10_ PFU for CYZs and wt Puerto Rico-2015 ZIKV, 1 to 3 log_10_ PFU for ChimeriVax-JE, and −1 to 2 log_10_ PFU for YF 17D). The Mock control group was inoculated with diluent, MEM supplemented with FBS. Mortalities are shown at doses determined by back-titration of the inoculates. LD_50_ values of CYZs vs. YFV-17D were statistically different (*P* = 0.012; Mann Whitney test).

Attenuation of CYZ-yfs and CYZ-hs variants was compared to the YF 17D benchmark and ChimeriVax-JE vaccine controls in a highly sensitive suckling mouse neurovirulence test that had been used extensively in the evaluation of other ChimeriVax vaccines^31^. Five-day old ICR suckling mice were inoculated with viruses by the intra-cerebral (IC) route and observed for signs of disease and mortality for 21 days. The CYZ variants caused only a few sporadic deaths at some of the doses tested (the intended doses were from −1 to 4 log_10_ PFU) which was similar to the highly attenuated ChimeriVax-JE control^32^. In contrast, YF 17D caused mortality in a dose-dependent fashion, as expected, with an IC LD50 of 0.44 log_10_ PFU (Fig. 2). Based on IC LD_50_ values, CYZ viruses (IC LD_50_ > 4 log_10_ PFU) are more than 3,500 times less neurovirulent in this model compared to the YF 17D benchmark (*P* = 0.012; Mann Whitney test) and are as highly attenuated as ChimeriVax-JE.

A wt ZIKV Puerto Rico-2015 strain was also used in this experiment. This virus has identical M and E protein sequences to CYZ (French Polynesia-2013) and it has not been mouse-adapted by serial brain-to-brain passages. It also caused low mortality (Fig. 2) and thus, this model is not optimal/applicable for the differentiation of LAVs from at least some, e.g., mouse-brain un-adapted, wt ZIKV strains. It should be noted however that pathology scores for CYZ viruses were lower than ZIKV Puerto Rico-2015 based on observed clinical signs, specifically, hunched posture, ruffled fur, weight loss and neurological manifestations (data not shown). On the contrary, a highly mouse brain-adapted Uganda-1947 (MR766) ZIKV strain administered at a 4 log_10_ PFU dose caused 100% mortality in a neurovirulence test in 3.5-week old ICR mice, which are less susceptible to IC infection than suckling mice (data not shown).

Immunogenicity of CYZ vaccine candidates was assessed in 3.5-week old ICR mice (8 animals per group) that were immunized on days 0 and 28 by the intra peritoneal (IP) route with 5 log_10_ PFU of CYZ-yfs and CYZ-hs variants, YF/Senegal-1894 and YF/Cambodia-2010 control chimeras, or wt ZIKV strains Mexico-2016 (I-44), Cambodia-2010, Senegal-1984 and Uganda-1947 [Two doses were used in this experiment because wt ZIKV was shown previously to not infect immunocompetent mice efficiently^33^ and thus we did not know whether CYZ and ZIKV controls would infect ICR mice to elicit a detectable immune response after one dose]. Neutralizing antibody responses were assessed after each dose to evaluate whether CYZ variants and other viruses would be immunogenic in ICR mice after one/two doses. All animals remained healthy throughout the study. After the first dose (Day 27), sera of mice immunized with CYZ variants and control chimeras displayed robust ZIKV-specific neutralizing antibody (N Ab) titers determined against both Puerto Rico-2015 and Uganda-1947 (MR766) ZIKV strains (PRNT_50_ GMTs 1282–7389 which were not statistically different against the two ZIKV strains; *P* > 0.5 by one-way ANOVA) (Table 1), with the CYZ-hs variant notably being the most immunogenic. wt ZIKV strains elicited variable N Ab titers which did not appear to correlate with their geographical location or year of isolation, or whether they were mouse-adapted (Table 1). After the second dose (Day 49), NAb GMTs increased to ≥10,000 in most groups, with the exception of sera from CYZ-yfs immunized mice titrated against ZIKV Uganda-1947 (GMT 2,000) and sera from ZIKV Uganda-1947 immunized mice titrated against ZIKV Puerto Rico-2016 (GMT 1,047).

**Table 1:** Immunogenicity of a single dose of CYZ variants and control viruses in immunocompetent ICR mice*.

Immunogen	PRNT_50_ GMT ± SD against ZIKV Puerto Rico-2015	PRNT_50_ GMT ± SD against ZIKV Uganda-1947
CYZ-yfs	1868 ± 1739	1282 ± 1165
CYZ-hs	7389 ± 8630	3774 ± 1796
YF/Cambodia-2010 contr.	2998 ± 1480	1562 ± 1040
YF/Senegal-1984 contr.	1421 ± 1352	2079 ± 1701
wt ZIKV Mexico-2016	727 ± 1956	392 ± 531
wt ZIKV Cambodia-2010	117 ± 55	329 ± 290
wt ZIKV Senegal-1984	2558 ± 3980	2528 ± 1607
wt ZIKV Uganda-1947	160 ± 435	1551 ± 722
Mock (MEM)	<100	<100

*PRNT_50_ titers were determined on Day 27 against wt ZIKV Puerto Rico-2015 and Uganda-1947 strains. The difference in titers against the two ZIKV strains was not statistically significant (*P* > 0.5; one-way ANOVA).

To evaluate the protective efficacy conferred by CYZ immunization, a pilot experiment was performed in naïve ICR mice to establish a ZIKV challenge model. Approximately 6-month old ICR mice were inoculated by the intravenous (IV) route with 2 log_10_ PFU of either Uganda-1947 (MR766) or Puerto Rico-2015 wt ZIKV strains. Viremia was measured in sera on Days 1, 2, and 5 post-inoculation. Interestingly, despite the fact that the Uganda-1947 (MR766) strain is highly mouse-adapted, no animals that received this virus showed viremia detectable by RT-qPCR. In contrast, 4 out of 5 mice that received the Puerto Rico-2015 strain showed detectable viremia (viral RNA) on Days 1 and 2 in the range of 1.1 × 10^3^ to 2.8 × 10^4^ genome equivalents (GE)/ml. In order to assess the protection mediated by CYZ, ICR mice that were immunized with 2 doses of CYZ-hs variant were challenged by the IV route with Puerto Rico-2015 ZIKV at 7 months post-immunization. No post-challenge viremia was detected by RT-PCR in CYZ-hs immunized animals indicative of a long-term protection, while 3 out of 6 mock-immunized mice developed viremia detected on Days 1–3 after challenge at titers up to 5.9 × 10^3^ GE/ml.

### CYZ attenuation, immunogenicity and protective efficacy in IFN-α/β receptor-deficient A129 mice following peripheral inoculation

A129 mice are an established, highly sensitive model for ZIKV viscerotropism and have been shown to succumb to ZIKV disease in an age-dependent fashion. Specifically, 100% mortality has been documented in 3-week old A129 mice, while 5-week old mice were partially susceptible and all 11-week old mice studied survived peripheral ZIKV infection^33^. ZIKV was also demonstrated to elicit a robust N Ab response in A129 mice^16^. Therefore, we used A129 mice to evaluate the attenuation, immunogenicity and efficacy of CYZ-hs candidate.

**Figure 3:**
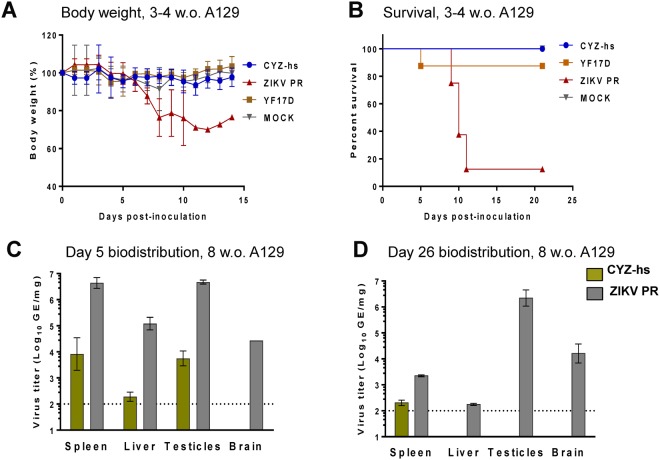
CYZ-hs attenuation in A129 mice. (**A**,**B**) 3–4 Week old A129 mice were inoculated SC with 5 log_10_ PFU of CYZ-hs, ZIKV Puerto Rico-2015, YF 17D or Mock (MEM diluent), and body weight (**A**) and survival (**B**) were monitored. Differences between the ZIKV and all other groups were statistically significant, *P* = 0.0002 by Log-rank (Mantel-Cox) test. (**C**,**D**) 8-Week old A129 mice were inoculated SC with 5 log_10_ PFU of CYZ-hs or ZIKV Puerto Rico-2015 viruses, and viral RNA loads in indicated organs were determined by RT-qPCR on Days 5 (**C**) and 26 (**D**). Differences between CYZ-hs and ZIKV were statistically significant (*P* < 0.0001, two-way ANOVA).

To measure peripheral virulence, young 3-4-week old A129 mice received 5 log_10_ PFU of CYZ-hs variant, ZIKV Puerto Rico-2015 or YF 17D by the subcutaneous (SC) route. ZIKV Puerto Rico-2015 induced neurological signs of infection, a significant reduction in body weight (Fig. 3A) and 87.5% mortality (Fig. 3B). In contrast, CYZ-hs virus was found to be highly attenuated as no weight loss, sickness or deaths were observed, as was seen in the mock-infected control group. YF 17D was also found to be highly attenuated in this model [differences between the ZIKV vs. all other groups were statistically significant, *P* = 0.0002 by Log-rank (Mantel-Cox) test] (Fig. 3).

Tissue tropism (biodistribution), which is another measure of attenuation, as well as immunogenicity and protective efficacy of vaccine candidates, were examined in older, 8-week old A129 mice. Following SC inoculation of animals (both males and females) with 5 log_10_ PFU of CYZ-hs or ZIKV Puerto Rico-2015, viral RNA loads were determined on Days 5 and 26 in the spleen, liver, brain and testicles (of males) using RT-qPCR in 3 animals per group/time point. On Day 5, high viral loads of ZIKV Puerto Rico-2015 were observed in all of these organs, with RNA concentrations of ~4.5–6.5 log_10_ GE/mg of tissues obtained. In contrast, CYZ-hs was significantly attenuated as viral loads were ~2.5 orders of magnitude lower in the spleen, liver and testicles, and importantly, no CYZ-hs virus was detected in the brain (Fig. 3C). On Day 26, ZIKV Puerto Rico-2015 was detectable in all the organs, with particularly high RNA loads in testes and brain (~6 and 4 log_10_ GE/mg, respectively), while CYZ-hs RNA was only detectable at low concentration in the spleen (Fig. 3D). Differences in CYZ-hs and ZIKV RNA loads were statistically significant on both days (*P* < 0.0001, two-way ANOVA). All mice remained healthy throughout this experiment.

In the inoculated 8-week old A129 mice, one dose of CYZ-hs variant elicited high N Ab responses measured in sera on Day 26 against both Puerto Rico-2015 and MR766 ZIKV strains (PRNT_50_ GMTs 1,354 and 2,995, respectively) (Table 2). Immunogenicity of CYZ-hs was comparable to that of the Puerto Rico-2015 virus. Immunogenicity of CYZ-dg and CYZ-dC variants with additional attenuating genetic modifications (with de-glycosylated E and 3-amino acid deletion in C, respectively) were also examined in this experiment. The modifications reduced neutralizing titers several-fold compared to CYZ-hs, although the variants remained rather immunogenic, with PRNT_50_ titers in the hundreds and considered to be at protective levels (Table 2). The titers determined against the two ZIKV strains were not statistically different in all groups (*P* > 0.5; one-way ANOVA).

**Table 2:** ZIKV-specific neutralizing antibody responses in A129 mice*.

Immunogen	PRNT_50_ GMT ± SD against ZIKV Puerto Rico-2015	PRNT_50_ GMT ± SD against ZIKV Uganda-1947
CYZ-hs	2284 ± 885	2995 ± 2701
CYZ-dC (3-a.a. deletion in C)	480 ± 420	850 ± 227
CYZ-dg (de-glycosylated E)	353 ± 210	708 ± 281
wt ZIKV Puerto Rico-2015	3900 ± 2573	3792 ± 9823
Mock (MEM)	<20	<20

*8-week old A129 mice were immunized SC with 5 log_10_ PFU of viruses and PRNT_50_ titers were determined in sera on Day 26 against wt ZIKV Puerto Rico-2015 and Uganda-1947 strains. The difference in titers against the two ZIKV strains was not statistically significant (*P* > 0.5; one-way ANOVA).

**Figure 4:**
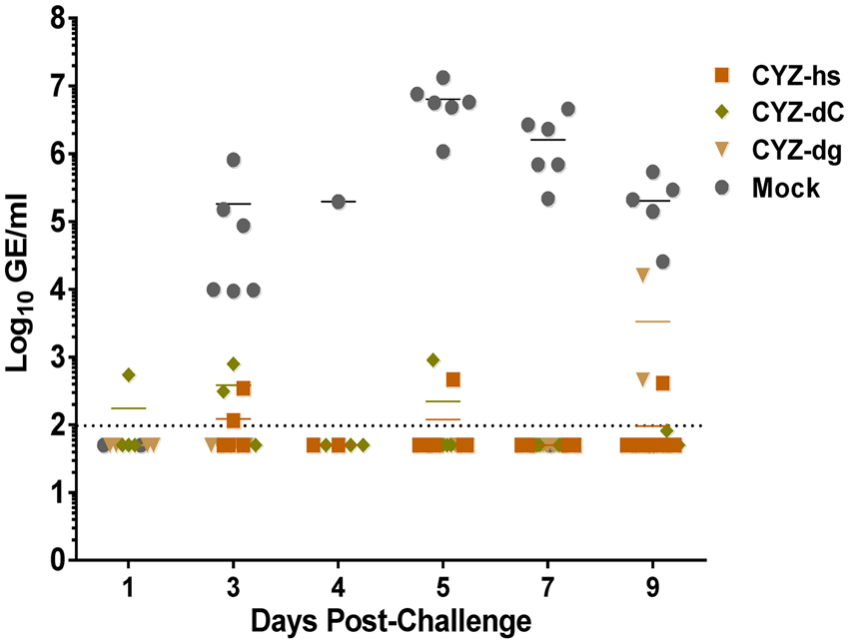
Protection of CYZ-immunized A129 mice from ZIKV challenge. 8-week old A129 mice were immunized SC with 5 log_10_ PFU of CYZ variants and challenged on Day 31 by SC route with 3 log_10_ PFU of wt ZIKV Puerto Rico-2015. Post-challenge viral RNA in sera (viremia) was measured by RT-qPCR. For Mock vs. CYZ groups on Days 3–9, P = 0.0002; one-way ANOVA.

To assess protection, mice were challenged SC on Day 31 with 3 log_10_ PFU of wt ZIKV Puerto Rico-2015. Post-challenge viremia (viral RNA) was measured by RT-qPCR in sera collected on Days 1, 3, 4, 5, 7 and 9. CYZ-immunized animals were protected as only very low RNA levels were detectable in some animals, which was not unexpected given the high sensitivity of RT-qPCR that could allow for detection of non-infectious (neutralized) viral particles, including from the challenge inoculate. In contrast, high post-challenge viremia was observed in mock-immunized controls peaking at ~7 log_10_ GE/ml on Day 5 (for Mock vs. CYZ groups on Days 3–9, *P* = 0.0002 by one-way ANOVA) (Fig. 4).

### CYZ attenuation *in vitro* in human neuronal cell culture models

**Figure 5:**
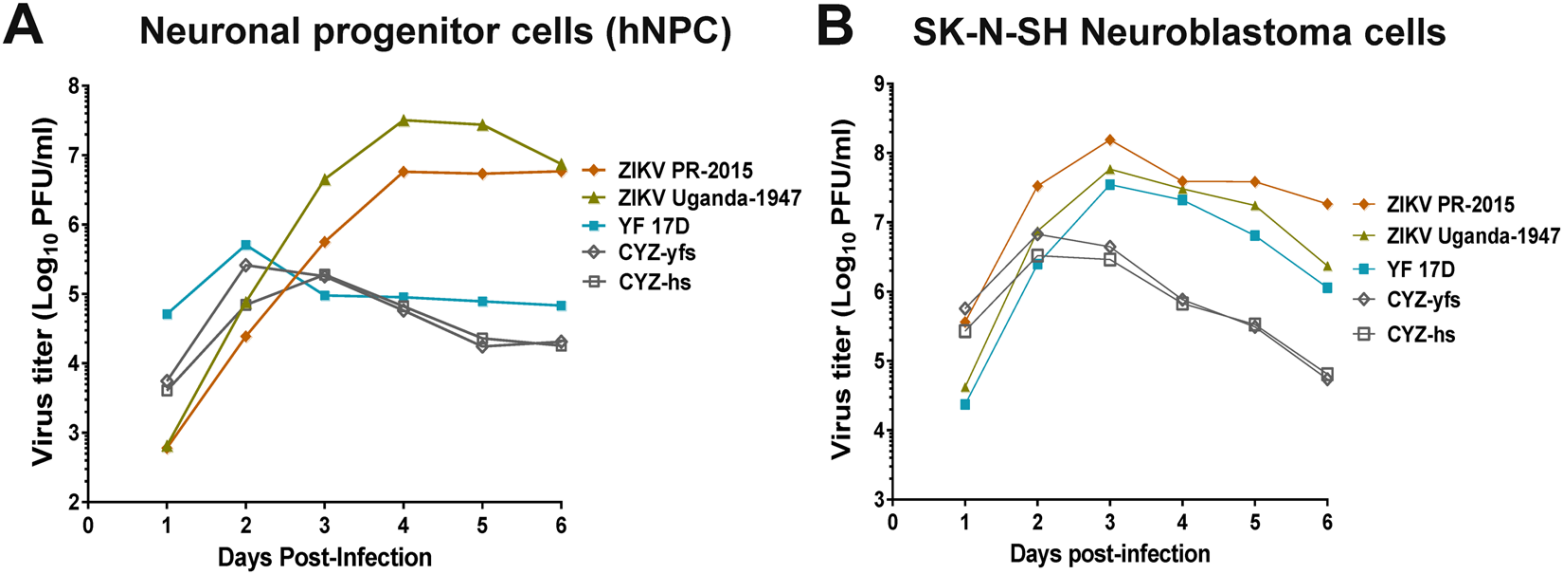
Replication of CYZ-yfs and CYZ-hs variants, and wt ZIKV (Puerto Rico-2015 and Uganda-1947) and YF 17D controls in (**A**) human neuronal progenitor cells (hNPC) and (**B**) human neuroblastoma SK-N-SH cells. MOI 0.01 PFU/cell. Representative data from one of 2 independent experiments are shown.

Attenuation in human neuronal cells was evaluated by comparing growth curves of CYZ-yfs and CYZ-hs and ZIKV Puerto Rico-2015 and Uganda-1947 strains, as well as YF17D, in human neuroblastoma (SK-N-SH) and human neuronal progenitor cell (hNPC) cultures at MOI 0.01 PFU/cell. The wt ZIKV strains grew efficiently in both hNPCs and neuroblastoma cells reaching peak titers of ~7 to >8 log_10_ PFU/ml (Fig. 5A and B, respectively). The YF 17D control was significantly attenuated compared to ZIKV for replication in hNPCs but not in neuroblastoma cells. Both CYZ-yfs and CYZ-hs variants exhibited a significant, ~100- and 40-fold reduction in peak titers in hNPCs and neuroblastoma cultures, respectively, compared to wt ZIKVs (Fig. 5). No significant differences in the induction of cytopathic effect (CPE) by the viruses were observed in both cell lines.

## Discussion

In this study, we applied the ChimeriVax approach to the development of a chimeric LAV candidate vaccine against Zika. The main advantage of LAVs is that they offer the possibility of enabling durable protection after a single dose without any adjuvant, similar to the empirically attenuated YF 17D vaccine. The development of an empirically attenuated ZIKV-based vaccine is not considered practical because of the unique features of ZIKV replication and pathology in humans, particularly the congenital Zika syndrome (microcephaly and other teratogenic effects) and sexual transmission, and the difficulty to establish reliable translational models to ensure safety of empirical LAV candidates, e.g. for the fetus in pregnant women. The ChimeriVax approach is one of the rational LAV approaches to a Zika vaccine that are currently being pursued (e.g.^16,17,34^).

While the main focus of this study was on the development of a Zika vaccine, our experiments yielded interesting information on flavivirus molecular biology, specifically the effect of C/prM signalase cleavage on virus replication. It has been proposed that a relatively inefficient signalase cleavage at this junction (downstream from a viral protease cleavage in C) facilitates the incorporation of nucleocapsid during virus budding and thus is an important prerequisite for efficient replication of flaviviruses. Enhancement of this cleavage in YF 17D was shown to abrogate replication^20–22^. In ZIKV however, the C/prM signalase cleavage predicted score is rather high in comparison to YF 17D. Furthermore, increasing the cleavage probability by replacing the YF 17D specific prM signal in CYZ-yfs chimera with a hybrid YF 17D/ZIKV prM signal unexpectedly resulted in a CYZ-hs variant which replicated more efficiently than CYZ-yfs in Vero cells. This indicates that the requirement for inefficient prM signalase cleavage is not necessarily true for all flaviviruses. However, the predicted cleavage efficiencies should be confirmed experimentally in further studies.

The CYZ-hs variant grew to the highest titers in Vero cells among other variants (e.g., over 8 log_10_ PFU/ml at MOI 0.001 PFU/cell) which is an important consideration for commercial manufacturing. For this reason, as well as its favorable attenuation profile and the observation that it elicited the highest immune responses in mice, we selected CYZ-hs as the lead vaccine candidate.

To ensure safety of flavivirus LAVs, neurovirulence in mice and non-human primates (NHP) should not exceed that of YF 17D virus which is used as a standard benchmark comparator. YF 17D is neurovirulent for immunocompetent mice, while the previously constructed ChimeriVax-JE, -WN and -DEN types 1–4 vaccines were shown to not be neurovirulent in adult mice, although a degree of dose- and age-dependent neurovirulence was observed in the more sensitive suckling mice at IC doses several orders of magnitude higher than YF 17D^9,11,23,32^. In 5-day old immunocompetent (ICR) suckling mice, IC inoculation with CYZ-hs resulted in few deaths and observed clinical signs (similar to ChimeriVax-JE control), with the IC LD_50_ > 4 log_10_ PFU, and the chimera was more than 3,500 times less neurovirulent than YF 17D (IC LD_50_ of 0.44 log_10_ PFU), thus satisfying the requirement of being highly attenuated in this standard assay. However, this test was not suitable to clearly differentiate CYZ-hs from a mouse-unadapted ZIKV Puerto Rico-2015 control that also caused low mortality. This result was not unexpected as most wt ZIKV isolates are not neurovirulent in mice unless they are mouse brain-adapted by serial brain-to-brain passages^35^. For instance, we have found that the mouse brain-adapted Uganda-1947 strain was lethal in 3.5-week old ICR mice (100% mortality at IC 4 log_10_ PFU dose).

A more clear differentiation of CYZ-hs (and CYZ-yfs) versus wt ZIKV with respect to replication in neuronal cells was obtained by comparing viral growth *in vitro* using human neuroblastoma and hNPC cultures. Replication in neuroblastoma cells is considered to be a predictor of pathology/attenuation of neurotropic flaviviruses^36,37^, and hNPCs and hNPC-derived cerebral organoids have been developed as models for studying ZIKV-induced pathology in the developing human brain^38–40^. The significant reduction in CYZ replication observed in these cells (40-100-fold lower peak titers) in comparison to both ZIKV Puerto Rico-2015 and Uganda-1947 strains is indicative of a significant attenuation of CYZ candidates for replication in human brain tissue. A reduced replication in the brain would reduce the risk of neuropathological adverse events after vaccination of adults and children and possibly brain abnormalities in the fetus of vaccinated pregnant women, and is thus a highly desirable characteristic in a Zika LAV.

Upon peripheral inoculation, wt ZIKV strains replicate in various organs and can cause disease in IFN receptor-deficient mice (A129, AG129, Ifnar) in a dose and mouse-strain and age-dependent fashion^33,41,42^. These mouse strains have been used in studies of attenuation/pathogenesis of other flaviviruses, e.g., YF 17D vaccine and dengue viruses^43,44^. Therefore, we further evaluated attenuation of the CYZ-hs variant in IFN-α/β receptor-deficient A129 mice following SC inoculation. CYZ-hs caused no signs of disease and no mortality in young (3–4 week old) A129 mice, which are susceptible to wt ZIKV^33^. This was in contrast to wt ZIKV Puerto Rico-2015 strain which induced clinical signs, weight loss and high mortality. Next, biodistribution was examined in older (8 week old) A129 mice. High levels of ZIKV Puerto Rico-2015 viral RNA (~4.5–6.5 GE/mg) were observed on Day 5 after SC infection in the spleen, liver, brain and testicles, while CYZ-hs RNA loads were at least 2.5 orders of magnitude lower. Moreover, in contrast to CYZ-hs, ZIKV Puerto Rico-2015 persisted, particularly in testicles and the brain, as evidenced by high RNA concentrations in these organs on Day 26. Thus CYZ-hs is highly attenuated for virulence and replication in organs in this model. The absence of CYZ-hs in testicles on Day 26 indicates that the chimera has lost the ability for long-term persistence in this organ, a feature that would reduce the risk of sexual transmission of CYZ vaccine virus after vaccination. Importantly, CYZ is not neuroinvasive as evidenced by the absence of viral RNA in the brain of mice on both Days 5 and 26. The absence of neuroinvasion is an important safety feature for all target populations for vaccine use, including adults and pediatric populations, and likely for vaccination of pregnant women. These data suggest that the NS proteins of the non-neuroinvasive YF 17D virus rendered the chimera unable to penetrate the brain in this model, as well as to persist in testes. A similar effect on neuroinvasiveness of NS proteins of a non-neuroinvasive virus was previously documented for a chimeric dengue 4/TBE LAV in that its wt TBE parent virus (donor of the prM-E genes) was lethal for mice after peripheral inoculation, while the chimera based on the backbone of the non-neuroinvasive dengue type 4 virus was not^45^. Our data in this study also suggest that NS proteins of ZIKV play a significant role in ZIKV tissue tropism/pathology.

Finally, a single dose of CYZ-hs was shown to be highly immunogenic in ICR and A129 mice, with PRNT_50_ titers in sera (GMTs) ranging from ~1,300 to 7,000. For comparison, it was observed earlier that Balb/c mice immunized with an inactivated Zika vaccine candidate with alum adjuvant developed N Ab titers of 15 as determined by micro-neutralization assay (albeit the values should be compared with caution due to differences in the assays used). This level of N Abs was sufficient to protect animals from surrogate challenge with ZIKV by the IV route^13^. Importantly, neutralizing titers in CYZ-immunized animals were equivalent when determined against both the recent American (Puerto-Rico-2015) and original African (Uganda-1947) isolates indicating that the CYZ vaccine is likely to be broadly protective against genetically distant ZIKV strains circulating in different geographical regions. Both ICR and A129 immunized mice were protected from challenge with ZIKV based on measuring viral RNA in post-challenge sera. ICR mice were protected from challenge at 7 months post-immunization suggesting that CYZ can provide durable protection.

The evidence described here indicates that the lead CYZ-hs candidate is highly attenuated while maintaining strong immunogenicity and ability to replicate efficiently in Vero cells. Taken together, these data support further studies in other models, including NHP, and suggest that the vaccine may be suitable for evaluation in human clinical trials and for commercial scale manufacturing. Standard NHP models used previously for other ChimeriVax vaccines should be largely sufficient to generate additional necessary preclinical evidence of safety and effectiveness of this LAV for adults (excluding pregnant women) and adolescents before they are sexually active. For sexually active adults, additional preclinical or clinical models may need to be applied to ascertain that there is no sexual transmission of a CYZ vaccine. Recently, a theoretical concern of enhancement of ZIKV infection or pathology by pre-existing antibodies against other flaviviruses was raised based on experiments *in vitro* and in small animals. There is no evidence of such enhancement occurring in humans and the available NHP data argue otherwise^46,47^. Nevertheless it may be necessary to demonstrate clinically that there is no enhancement of CYZ vaccine infection by cross-reactive anti-flavivirus antibodies and thus, that vaccine safety would not be compromised in pre-immune subjects. Finally, if the intended target population will include pregnant women, safety of a CYZ vaccine for the fetus will need to be demonstrated, preferably in an appropriate, reliable NHP model.

## Materials and Methods

### wt ZIKV strains and control vaccine viruses

The wt ZIKV strains used in this study were: MR766 (Uganda-1947), I-44 (Mexico-2016), FSS 13025 (Cambodia-2010), and DAK AR 41519 (Senegal-1984) obtained from Dr. Robert Tesh, World Reference Center for Emerging Viruses and Arboviruses (WRCEVA, UTMB, Galveston, TX), and PRVABC59 (Puerto Rico-2015) from the Centers for Disease Control and Prevention (CDC, Atlanta, GA). Viruses were propagated and titrated similarly to CYZ chimeras as described below. ChimeriVax-JE chimera was described previously^32^.

### Construction and *in vitro* characterization of CYZ variants

Insertion of ZIKV prM-E genes (French Polynesia-2013 strain; GenBank accession number KJ776791) into the YF 17D backbone in a low-copy number pBeloBac11-based single-plasmid vector^48^ and other genetic modifications were done using gene synthesis (by DNA2.0, Inc., Menlo Park, CA, or gene blocks by Integrated DNA Technologies, Skokie, IL) and standard cloning approaches. The prM-E genes from representative Asian (Cambodia-2010, GenBank accession number JN860885) and African (Senegal-1984, GenBank accession number HQ234501) ZIKV strains were also used for construction of control chimeras.

Resulting plasmids were linearized with the restriction enzyme *Xho*I and *in vitro* transcribed using Amplicap SP6 High Yield Message Maker kits (Cellscript, Madison, WI). *In vitro* transcribed RNA was then transfected into Vero cells to generate CYZ infectious viruses. All viral recoveries, further passages and growth curve analyses in Vero cells were performed using serum free VPSFM medium (11681–020; Gibco, Invitrogen Corporation, Carlsbad, CA) at 37 °C, 5.0% CO_2_. Virus stocks were supplemented with 10% sorbitol and stored at −80 °C. Titers of viruses were determined in Vero cells either using a standard plaque assay on Day 5 post-infection with crystal violet staining^24,32^ or by immunostaining of foci as previously described^49^ with anti-ZIKV E protein MAb 0502156 (Aalto Bioreagents, Dublin, Ireland) and anti-mouse IgG Fc-HRP conjugated secondary antibody (31437; Thermo Fisher Scientific, Waltham, MA). Genetic stability was confirmed by serial passage in Vero cells to passage 5 (P5) at controlled MOIs of 0.01–0.001 PFU/cell followed by RT-PCR and consensus sequencing of the prM-E genes and additional regions of the genome where needed, such as the C gene and 3′UTR of variants with modifications in these elements, or full genomes.

Attenuation of CYZ replication in human neuronal cells was assessed *in vitro* by analyzing growth curves in human neuroblastoma and hNPC cells and compared to wt ZIKV and YF 17D controls. Human SK-N-SH neuroblastoma cells (American Type Culture Collection/ATCC, Manassas, VA) were maintained in ATCC-formulated Eagle’s MEM (ATCC) supplemented with 10% FBS. hNPCs (ATCC) were grown in DMEM F12 medium with Neural Progenitor Cell Expansion kit supplements (ATCC). Cells grown in T-25 flasks or 6-well tissue culture plates (in the case of hNPCs) were infected with viruses at indicated MOIs and incubated at 37 °C, 5.0% CO_2_. Virus titers were determined in aliquots of media harvested daily.

### Mouse studies

All procedures were performed under approved IACUC protocols in accordance with the National Institutes of Health requirements for humane treatment of laboratory animals. Inoculation routes/doses, and procedure days were as described in Results.

### Mouse neurovirulence tests

Immunocompetent (ICR) mice were obtained from Charles River (Charles River Laboratories International, Inc., Wilmington MA, USA). 5-day-old suckling mice, in groups of 9 to 12 (1 dam per group; suckling mice randomized between groups) were inoculated by the IC route with wt ZIKV, CYZ, LAV and mock control at the indicated doses in 20 μl of MEM containing 0.25% human serum albumin using a 0.5-ml Hamilton Syringe and 27G ¼” microneedles. Inoculated mice were observed for 3 weeks for symptoms of encephalitis, including ruffled hair, hunched back, paralysis, and death. Moribund animals were humanely euthanized.

### Evaluation of immunogenicity in immunocompetent mice, and attenuation and immunogenicity in immunodeficient A129 mice

3.5-week-old ICR mice were immunized twice (Days 0 and 28) by the IP route with the indicated test articles and controls (8 animals per group). ZIKV-specific neutralizing antibody titers were determined after both the first and second doses (Days 27 and 53) in heat-inactivated serum samples using a standard PRNT_50_ assay^49^ against wt ZIKV strains Puerto Rico-2015 and Uganda-1947 (MR766). Animals were challenged by the IV route at ~7 months post-immunization with 2 log_10_ PFU Puerto Rico-2015 ZIKV and post-challenge viremia was measured in serum on Days 1–5 by RT-qPCR using primers/probe specific for the ZIKV E gene.

To measure attenuation in immunodeficient mice, 3-4-week old A129 mice (IFN-α/β receptor deficient; Marshall BioResources, North Rose, NY, USA), which are susceptible to ZIKV, were inoculated with 5 log_10_ PFU of CYZ or indicated control viruses by the SC route followed by observation of neurological signs of infection, weight loss and mortality.

To assess tissue tropism as well as immunogenicity and protective efficacy, older, 8-week old A129 mice (both males and females) were inoculated SC with CYZ and control viruses. Biodistribution was determined on Days 5 and 26 by measuring viral RNA loads in the spleen, liver, brain and testicles using RT-qPCR in 3 animals per group/time point. ZIKV-specific antibody responses were measured in sera on Day 26 by PRNT_50_. Animals were challenged SC on Day 31 with 3 log_10_ PFU of wt ZIKV Puerto Rico-2015. Post challenge viremia was measured by RT-qPCR in sera collected on Days 1–9.

### Statistical analyses

Calculations of endpoint titers and statistical analyses were performed using GraphPad Prism 6 (GraphPad Prism Software, Inc., San Diego, CA).
